# Influence of habitat suitability and sex-related detectability on density and population size estimates of habitat-specialist warblers

**DOI:** 10.1371/journal.pone.0201482

**Published:** 2018-07-30

**Authors:** Óscar Frías, Luis M. Bautista, Francisco V. Dénes, Jesús A. Cuevas, Félix Martínez, Guillermo Blanco

**Affiliations:** 1 Department of Evolutionary Ecology, National Museum of Natural Sciences, CSIC, Madrid, Spain; 2 Department of Conservation Biology, Estación Biológica de Doñana, CSIC, Américo Vespucio s/n, Sevilla, Spain; 3 Department of Life Sciences, UD Ecology, University of Alcalá, Alcalá de Henares, Madrid, Spain; 4 Sociedad para la Conservación de los Vertebrados, Leganés, Madrid, Spain; Charles University, CZECH REPUBLIC

## Abstract

Knowledge about the population size and trends of common bird species is crucial for setting conservation priorities and management actions. Multi-species large-scale monitoring schemes have often provided such estimates relying on extrapolation of relative abundances in particular habitats to large-scale areas. Here we show an alternative to inference-rich predictive models, proposing methods to deal with caveats of population size estimations in habitat-specialist species, reed warblers (*Acrocephalus scirpaceus* and *Acrocephalus arundinaceus*). Reed warblers were only found in pure reedbeds within riparian woodlands or in riparian vegetation scattered within or around reedbed patches, as expected according to their habitat specialization. The proportion of individuals located in reedbed associated with lotic and lentic waters differed between species, and no reed warbler was recorded in reedbed located along dry streams. This indicates that microhabitat features or their effects on reedbed structure and other factors made a proportion of the apparently available habitat unsuitable for both warbler species. Most warblers detected were males performing territorial singing (females seldom sing and do not perform elaborate territorial song, and are undistinguishable from males by plumage). The regional population sizes of the warbler species (~4000 individuals of *A*. *scirpaceus* and ~ 1000 individuals of *A*. *arundinaceus*) were much smaller than those estimated for the same area by transforming relative abundance obtained at a national scale to population size through extrapolation by habitat at a regional scale. These results highlight the importance of considering the habitat actually used and its suitability, the manner of sex-related detection, population sex-ratio and their interactions in population estimates. Ideally, the value of predictive methods to estimate population size of common species should be tested before conducting large-scale monitoring, rather than *a posteriori*. Although logistically challenging, this can be achieved by designing monitoring programs including an intensive sampling of abundance in ad hoc reference areas of variable size.

## Introduction

Knowledge of bird population size is paramount to set conservation priorities and actions. Multi-species large-scale monitoring schemes have often provided these estimates relying on extrapolation of relative abundances in particular habitats to large-scale areas [1–3]. These estimates have been generally proposed without comparison to directly censused population size of particular species in reference areas, which often hampers an objective assessment of their accuracy [4–5]. While these shortcomings have been recognized for endangered species with comparatively small populations (e.g. [6–7]), they can be more frequent but rarely tested for common bird species due to the general difficulty in attaining a complete targeted census of entire populations. However, intensive sampling of individuals, territories and nests in reference areas of variable size can yield population size estimates as reliable and accurate as possible [8–9]. Although generally expensive and laborious, intensive sampling aimed to determine actual population sizes of habitat-specialist species can be logistically affordable when the target habitat occupies relatively small, discrete or localised areas.

Understanding the factors that determine site suitability for species occurrence is essential for inferential or predictive approaches based on large-scale extrapolation by habitat, because erroneously attributing unsuitable habitats as occupied can lead to misleading conclusions such as overestimation of population size or, given a specific abundance, underestimation of density [10]. In addition, sampling often also suffers from imperfect detectability and sex-related detection biases [9, 11]. Because females of many small passerines generally remain hidden during the breeding season and do not perform territorial singing activities, their presence often goes unnoticed and thus the sampling records and counts are underestimated and biased towards males [11,12]. These biases can have important implications when population size is estimated based on a mix of different types of detection data (visual and auditory) at variable proportions depending on habitat features or individual traits influencing detectability [13]. However, the mode of detection, and its frequency associated with particular sex-related activities (e.g. male territorial activities) are rarely recorded in large-scale multispecies monitoring programs. Therefore, accounting for sex-related detection mode and detectability coupled with knowledge of species-specific population sex-ratios owing to monogamous or polygamous reproductive strategies can contribute to more accurate population size estimates.

In this study, we present estimates of regional population sizes of two species of reed warblers based on intensive surveys and propose them as an alternative to large-scale predictive models based on extensive sampling and habitat extrapolation. We selected the Eurasian Reed Warbler *Acrocephalus scirpaceus* and the Great Reed Warbler *Acrocephalus arundinaceus* because they show a strict dependence on reedbed [14–15]. This habitat specialization allowed us to conduct focused intensive sampling throughout potential nesting habitat during the breeding season. We evaluated the frequency of the different modes of detection (auditory or visual). Because of its influence on size estimation of populations wherein territorial males are mostly detected, we assessed whether the sex-ratio can be used in population size estimates. We assessed whether suitable habitat can be distinguished from unsuitable habitat according to reedbed characteristic, specifically the presence of water, and especially from unused habitat (i.e. the matrix of riparian woodland and other vegetation types). Because the presence of water may depend on wetland type, we also tested whether the warbler’s occurrence differs between reedbed at lentic and lotic waters, and whether it differs between the two study species. We hypothesized that population size estimates of habitat-specialist warblers would be biased by neglecting habitat availability and suitability. This hypothesis predicts an overestimation of population size if abundance estimates obtained in suitable habitat is extrapolated to unused and unsuitable habitat over large areas. To test this prediction, our estimates were compared with previously proposed population size estimates obtained through extrapolation of relative abundance by habitat over large areas without considering sex-biased detectability and habitat availability and suitability.

## Material and methods

### Ethical statement

No specific permits were required for this study. The geographic coordinates of the study sites were provided in S1 Table. The species observed were protected but not endangered.

### Study species and study area

The Eurasian Reed Warbler (RW hereafter) and the Great Reed Warbler (GRW hereafter) are trans-Saharan migrant passerines with a widespread but fragmented breeding distribution in the Palearctic due to the strict dependence of reedbed on patchy marsh systems [14–15]. They are habitat specialists, establishing territories, foraging and nesting in reedbed of variable extent, either formed of monospecific or mixed patches dominated by reed (*Phragmites* sp.) and reedmace (*Typha* sp.) or patches of these plant species interspersed within a matrix of marshland and riparian vegetation depending on the region [16–19]. During the breeding period males sing intensively to defend territories and attract mates [14–15].

**Fig 1:**
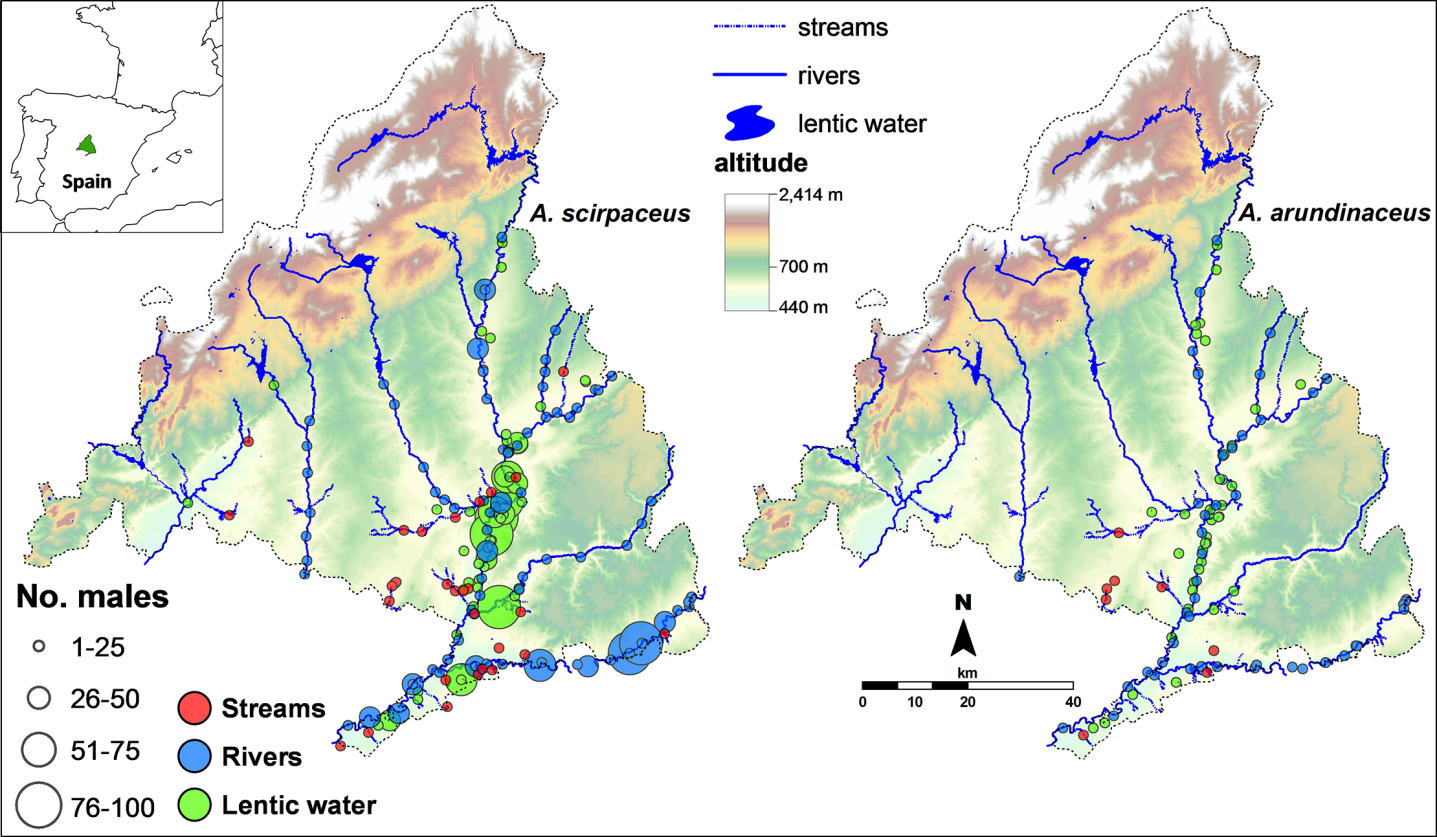
Study area. Madrid province, central Spain, showing the distribution of reed beds with presence of *Acrocephalus* warbler species (*A*. *scirpaceus* and *A*. *arundinaceus*) in rivers, streams and lentic water. Each circle represents a sampling locality, while circle size represents the number of territorial (singing) male warblers.

The study was carried out in wetlands (rivers, streams, lakes) of Madrid province (c. 8000 km^2^), central Spain (Fig 1). In 2010, we located and visited all places with marsh vegetation to determine the presence of reedbed. Marsh patches were located along 610 km of lotic water (rivers, wet and dry streams, channels of irrigated cultivations, including those delimiting the boundaries of the study area) and 125 km of lentic water (seasonal lakes, artificial ponds and flooded gravel pits) perimeters (Fig 1). Distribution of rivers and wetlands in the visited watersheds is detailed in S1 Table. The main habitat types identified were reedbed (*Phragmites australis*, *Typha angustifolia* and associated *Arundo donax*), riparian woodlands (*Populus alba* and *P*. *nigra*, *Fraxinus angustifolia*, *Ulmus minor*, *Salix* sp., *Tamarix* sp., *Rubus* sp.) and other habitats (dry and irrigated agricultural fields, urban, sub-urban, pinewoods and vineyards) where small reedbed patches were associated with permanent or temporarily flooded water courses. Due to the lack of a specific GIS layer for reedbed, we measured their extent as well as that of the marshland and riparian vegetation matrix separately, excluding free-water areas, using aerial photographs (Spanish Land Parcel Identification System, SIGPAC facility, Spanish Ministry of Agriculture and Environment) available online (http://www.mapa.es/sig/pags/sigpac/intro.htm).

### Field procedures

In May-July 2010, we conducted intensive surveys covering the whole area of reedbed along rivers and within wetlands in Madrid province, including small patches interspersed within marsh and riparian vegetation. We employed 53 days of field work with 1–5 people, totalling 125 person-days. The surveys were conducted during the breeding period in the study area and avoided migratory periods [20]. We visited all potentially suitable sites for the presence of warblers, including all sites where their presence was previously recorded [21–22].

We surveyed transects between sunrise and midday, avoiding unfavourable meteorological conditions (such as rain and strong wind) that reduce aural and visual detection as well as warbler singing activity. The observation effort in each stretch depended on its length and the number and size of reedbed patches. The habitat configuration and the relatively narrow band of reedbed along rivers and around lentic waters allowed us to accurately locate and quantify the number of territorial males in a given reedbed patch owing to their singing activity. Therefore, the rationale of our intensive approach was to reach a complete census, so we adjusted the census effort to the particular habitat features of each reedbed patch to attempt reaching counts of territorial males as close as possible to the real population size, as if effort was unlimited [5]. The average width of reedbed in each censused stretch was calculated by dividing the total area of reedbed by the transect length. The relatively small widths of the rivers in our study area (mean ± SD: 21.4 ± 10.5 m, *n* = 141 stretches) allowed us to survey both margins in a single transect in most cases; drainage channels of irrigated crops sometimes including narrow strips of marsh vegetation were also surveyed longitudinally. Lentic waters including gravel pits, ponds and temporal and artificial lakes were surveyed with a perimeter walk-around with transversal transects on the wider reedbed to cover all reedbed and the marshland matrix (mean width: 69.9 ± 52.9 m, mean perimeter length: 1.9 ± 1.6 km, *n* = 65 wetlands).

In each survey site, we conducted census on transects by slowly (2–3 km/h) walking along rivers and around marshland margins, with incursions inside large reedbed associated with lentic water areas. Most transects were approximately straight lines along the rivers and streams, which included most reedbed in the study area. Transects were established on all reedbed patches within each habitat stretch, delimited according to geographical and physiognomic features, i.e. river courses, orientation, interruptions due to geographical features, etc. We systematically and intensively searched for warblers, with a variable number of stops of variable duration (5–10 min) to locate all individuals in the vicinity of each stopping point; this allowed us to locate each approximate bird position and movements during each observation period in an attempt to avoid double-counting, similarly to the method used for territory mapping [8]. The time devoted to each stop varied according to the length, extent and number of reedbed patches interspersed within the marshland vegetation matrix. We recorded the activity of warblers at first detection, categorized as performing territorial-mating singing (only males), performing distress or alarm calls (both sexes) or detected visually standing in the vegetation or flying without emitting any sound (both sexes). We tested whether the two warbler species differed in the use of lentic and lotic habitat by using Chi-squared test with Yates correction. We considered the total number of individuals and the number of territorial males of each species detected in each habitat. We also attempted to record the distance from the transect line to every detected warbler to estimate densities; however, because most detections were of vocalising birds whose position could not be determined accurately, and because lotic reedbed strips were too narrow and the habitat gradient was parallel to the transect line, we could not reliably estimate densities based on recorded distance data.

To test for the potential error in the number of territorial males obtained by single counts within each habitat stretch, we repeatedly visited and surveyed a sample of stretches (*n* = 9) along different rivers with reedbed (Henares, Tajo and Jarama rivers) and around a lake (*n* = 1, Laguna de San Galindo in the Tajuña river basin, see S1 Table for the location coordinates). The censuses were carried out following the same methodology described above on two different days, where the second census was carried out one up to 23 days later than the previous one in the same stretch (mean ± SD = 6.4 ± 5.5, *n* = 10). Overall, we conducted repeated censuses over 28.46 km of habitat stretches (27.96 km along rivers and 0.5 around the censused lake), thus totalling about 57 km of census. The mean ± SD length of the stretches censused twice was 2.9 ± 1.9 km (*n* = 10).

### Population size estimates

Detectability of many bird species during the breeding season may be biased toward males, because their activity pattern makes them more available to detection with an evident risk to underestimate females or non-breeding individuals [23]. Warbler females seldom sing, they exhibit cryptic behaviour in the mating season and are undistinguishable from males by size or plumage [14–15]. However, most males attempt to attract females by singing with variable success in establishing single (monogamous) or multiple (polygynous) territories, or no success in the case of non-breeding males remaining as floaters [14–15]. Given that sex-ratio of reed warblers in the breeding season can vary between years, population size should be ideally determined by using values recorded in the same area and breeding season in which the censuses are conducted. Therefore, the estimates of abundance of singing males were multiplied by the average inter-annual sex-ratio of adult warblers during the breeding seasons of 1995–2003 (see details in [24]) to estimate the total population size. In this form, we were able to calculate a broad range of likely population sizes using minimum and maximum sex ratios [24]. However, because the study was conducted in a single breeding season, we cannot discard that the sex-ratio in this season was out of the range used as reference. The number of territorial males per location (shown in S1 Table) allows testing for accuracy of counts in particular sites by independent observers and the assessment of population trends by conducting partial or complete censuses in the future [25]. Finally, bird density was computed as the total number of warblers or territorial males per reedbed area (in hectares), considering both the whole extent of reedbed patches (available habitat) and that of patches used by each warbler species (suitable habitat).

## Results

### Habitat availability and use

Overall, we recorded 3176 RWs and 424 GRWs through direct counts (including singing males, unsexed individuals, etc.). The available reedbed occupied 414.3 ha, distributed in 4549 discrete patches with a patch mean (± SD) area of 0.09 ± 0.46 ha. Mean (± SD) distance between reedbed patches was 56.7 ± 48.7 m. Most individuals were located in pure reedbed (98.2% of RWs, *n* = 3018 and 99.7% of GRWs, *n* = 397) or in riparian vegetation interspersed within or around small reedbed patches (1.8% of RWs, *n* = 3018, and 0.3% GRWs, *n* = 397). The remaining individuals were not assigned to any of the cited habitat categories, although they were always detected in pure or mixed reedbed. No warbler was detected in the 3220.3 ha matrix of pure riparian woodland in which reedbed patches were interspersed.

About 65% of RWs (*n* = 3176) and 48% of GRWs (*n* = 424) were located in reedbed along rivers and streams (lotic habitat), while the remaining individuals were located in reedbed around ponds and flooded gravel pits (lentic habitat). This indicates between-species differences in the use of reedbed occurring at lotic *vs*. lentic waters (Chi-squared test with Yates correction χ^2^_1_ = 42.45, *P* < 0.0001). The proportions of territorial males located in reedbed occurring at lotic waters *vs*. lentic waters were 62.7% *vs*. 37.3% (*n* = 2282) for RWs and 48.7% *vs*. 51.3% for GRWs (*n* = 343); this difference being statistically significant (*χ*^2^_1_ = 15.84, *P* < 0.0001).

### Mode of detection

**Fig 2:**
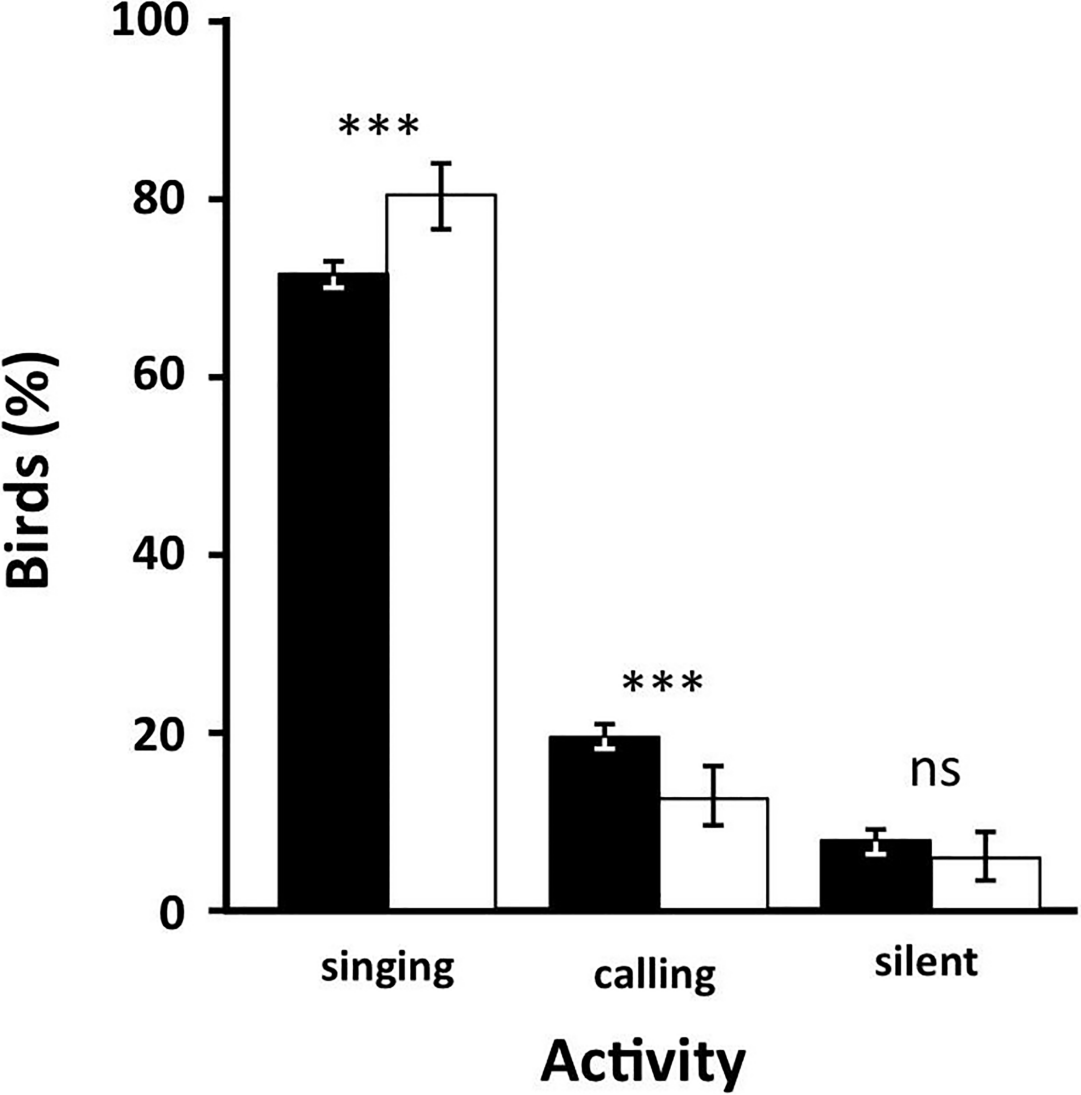
Detection mode. Relative frequency (%) of warblers recorded by each detection method, including territorial singing (only males), calling (males and females) and detected visually (males and females). Great Reed warbler males (open bars) were detected significantly more often singing than Eurasian Reed warbler males (filled bars). ***: *P* < 0.001; ns: *P* > 0.050. Error bars show the 95% confidence intervals of each percentage.

Most warblers detected were singing males (71.9% of RWs, *n* = 3176, 80.9% of GRWs, *n* = 424), while a lesser proportion of birds were first detected calling or visually, either perching or flying (Fig 2). GRWs were recorded singing in a greater proportion than RWs (*χ*^2^_1_ = 15.9, P < 0.0001, Fig 2). Because territorial males detected by song accounted for most contacts (Fig 2), we focused on them as the basis for population size estimates.

### Count error

The number of territorial males recorded in repeated counts of the same reedbed stretches did not differ for RWs (Wilcoxon matched-pair test, *z* = -1.16, *P* = 0.25) and GRWs (*z* = -1.16, *P* = 0.25). Pooling all stretches, the overall differences in the number of males recorded between the first (RW = 86, GRW = 7) and the second counts (RW = 81, GRW = 7) represent 5.8% and 0.0% for RW and GRW respectively. The difference in the number of males recorded between censuses for the same stretch was not correlated with the number of days elapsed between censuses (Spearman correlation, RW: *r*_s_ = 0.18, *P* = 0.63; GRW: *r*_s_ = 0.50, *P* = 0.14, *n* = 10) nor with the length of the stretches (RW: *r*_s_ = 0.05, *P* = 0.90; GRW: *r*_s_ = 0.59, *P* = 0.08, *n* = 10).

### Population size and density

The counts of territorial males, the mean sex-ratio in the population, the estimated number of females, and the total population size in each wetland type and its range according to the inter-annual minimum and maximum sex-ratio are shown in the Table 1, while the density values are shown in Table 2. Lentic waters, especially rivers with permanent flooding water showed about 63% of RW population, while lentic and lotic waters showed a similar proportion around the half of the GRW population size (Table 1). Overall, population density was about between three and four times greater in RW than in GRW, both considering available and suitable habitat (Table 2). The number of territorial males per location is shown in S1 Table.

**Table 1:** Counts of territorial (singing) males of *A*. *scirpaceus* and *A*. *arundinaceus* according to habitat type in Madrid province, central Spain.

	Territorial males	Estimated number of females			
	counts	mean	minimum	maximum	Population size (range)
*Acrocephalus scirpaceus*					
Rivers (210.0 ha)	1260	742	435	1260	2002 (1695–2520)
Flowing streams (36.5 ha)	171	101	59	171	272 (230–342)
Dry streams (31.9 ha)	0	0	0	0	0
Ponds and lakes (135.9 ha)	851	501	293	851	1352 (1144–1702)
Total	2282	1344	787	2282	3626 (3069–4564)
Sex ratio		1.70	2.90	1.00	
*Acrocephalus arundinaceus*					
Rivers (210.0 ha)	159	274	187	398	433 (346–554)
Flowing streams (36.5 ha)	17	29	20	43	46 (37–60)
Dry streams (31.9 ha)	0	0	0	0	0
Ponds and lakes (135.9 ha)	167	288	197	418	455 (364–585)
Total	343	591	404	859	934 (747–1202)
Sex ratio		0.58	0.85	0.40	

The extent of reedbed is shown in parentheses for each habitat type. The total counts of singing males were divided by the average inter-annual sex-ratio (males:females) of adult RW (*n* = 1372) and GRW (*n* = 274) captured during the breeding season of 1995–2003 in the study area (data extracted from [24]) to estimate female abundance. Population size estimates are the sums of the counts of territorial males plus the mean number of females; the range of the population size was calculated by considering the count of territorial males divided by the minimum and maximum inter-annual sex-ratio.

**Table 2:** Estimates of population density of *A*. *scirpaceus* and *A*. *arundinaceus* according to available and suitable habitat in Madrid province, central Spain.

		density (individuals/ha)	
	Number of warblers	available habitat	suitable habitat
*Acrocephalus scirpaceus*			
Reedbed extension (ha)		414.28	407.96
All detected individuals	3176	7.67	7.78
Count of territorial males	2282	5.51	5.59
Total population size (range)	3626 (3069–4564)	8.75 (7.40–11.02)	8.89 (7.52–11.19)
*Acrocephalus arundinaceus*			
Reedbed extension (ha)		414.28	303.33
All detected individuals	424	1.02	1.40
Count of territorial males	343	0.83	1.13
Total population size (range)	934 (747–1202)	2.25 (1.80–2.90)	3.08 (2.46–3.96)

Density was estimated for (i) all detected individuals (including all individuals recorded by all detection methods), (ii) count of territorial (singing) males and (iii) total population size (including singing males and the number of females estimated by using population sex ratio, and its range). The numbers of warblers were divided by the extent of the available and suitable habitat for each species to calculate density. The available (with and without warbler’s presence) and suitable habitat (with warbler’s presence) differed within and between species because of differences in the extent of the reedbed patches where we found warbler of each species (see the text for details).

## Discussion

Distinguishing suitable from unsuitable and unused habitats is fundamental for assessing population status and dynamics of birds and other organisms [26–27]. Specifically, it can become crucial when extrapolations of relative abundance are applied to habitats within large areas to estimate the population size of common species [3, 28–29]. As predicted, the regional population sizes of two species of warblers estimated in this study (~4000 RWs and ~ 1000 GRWs) were much smaller than those estimated for the same area by transforming relative abundance obtained at a national scale to population size through extrapolation by habitat at a regional scale (~23000 and ~17000 individuals respectively, [30]). Such large estimates based on extrapolation by habitat are likely positively biased by non-random selection of sampling units inflating density, coupled with density extrapolation to unavailable and unsuitable habitat (see also [4–5]). The alternative stating that these huge differences in population size could be due to extreme environmental conditions during the study breeding season was not probable according to the values of long time series of meteorological data from the study area consulted in http://www.aemet.es/.

Reed warblers were only found in pure reedbeds within riparian woodlands or in riparian vegetation scattered within or around reedbed patches, as expected by their habitat specialization [14–15]. The proportion of individuals located in reedbed associated with lotic and lentic waters differed between species, which suggests differences in microhabitat use [14–15]. In addition, the fact that no reed warbler was recorded in several reedbed patches, especially those located along dry streams indicates that microhabitat features such as the lack of water or its effects on reedbed structure and other factors (e.g. invertebrate availability) made a proportion of the apparently available habitat unsuitable for both warbler species [14, 18, 31].

The consideration of particular habitats used by target species or communities often conflicts with the confounding reduction of environmental complexity into habitat classification categories based on vegetation and landscape features available in large-scale geographic information systems (GIS) databases, which are extensively used in predictive habitat modelling research (e.g. [26, 32]). This simplification of the environmental complexity can become problematic when habitat extrapolation of relative abundance is applied to habitats or vegetation types not specifically included in the available databases, but pooled together with other habitat categories. As it occurs in our case study, patchy or low-extent habitats, such as reedbed, are rarely included as discrete variables in public GIS databases (for Spain see http://www.idee.es/web/guest/inicio), thus precluding their application to the study of density and abundance of species specialized in this habitat. Here, we showed that distinguishing suitable habitat (i.e. wet reedbed) from unsuitable habitat (i.e. dry reedbed), and especially from unused habitat (i.e. the matrix of riparian woodland and other vegetation types) becomes essential for population size estimation of reed warblers. In addition, microhabitat structure (i.e. linked to lentic or lotic waters) had a considerable influence on population abundance of each species of reed warblers. However, such microhabitat requirements are frequently disregarded in population size inferences based on extrapolation over large scales [4–5], which often assume that average density obtained by sampling the available habitat mirrors that of the suitable occupied habitat [30]. Such estimates can be further influenced by the suitability and quality of the sampled units, as determined by their spatial distribution according to the available habitat in large-scale monitoring studies. These programs have been generally conducted by volunteer observers [33], which can select sampling units based on their subjective perception, experience and preference, although it depends on programs and countries (for Spain see http://www.seo.org/?p=5943). This can result in unintended biases towards “good habitat” for birds thus inflating relative abundance of the target species. To avoid this bias, large-scale monitoring of common species should rely on sampling unit locations determined at random by means of automatic methods, rather than on volunteer selection. In the case of habitat-specialist species, these assessments would require sampling units randomly selected automatically but also considering suitable habitat distribution.

Auditory detection involving territorial males generally accounts for a great proportion of records of small passerines in transects and point counts [13, 34, 35]. This is mostly due to the intense male singing activity coupled with the difficulty of visual detection when birds are silent and in dense vegetation during the breeding season. In this study, most warblers detected were males performing territorial singing. Thus, the habitat configuration and the relatively narrow band of reedbed along rivers and around lentic waters allowed us to accurately locate and quantify the number of territorial males in a given reedbed patch owing to their singing activity. This highlights the crucial importance of the often-neglected mode of detection and its expected proportions among the records obtained in studies estimating detectability-based abundance and population size of common bird species (http://www.seo.org/?p=5943, [30]).

The discontinuous distribution of small patches of suitable reedbed, the associated relatively low abundance of reed warblers, the continuous and conspicuous singing activity of males, and the intensive fieldwork effort allowed us to be confident that we were detecting most territorial males. This was supported by the low difference in the number of males recorded in repeated counts in particular reedbed stretches. Because in this study population estimates were exclusively based on records of singing males regardless of their success of establishing territories and attracting mates (thus including singing floaters), overall population size was estimated by considering a proportional number of females as determined by population sex-ratio. This trait can be particularly influential in population size estimates based on territorial males of polygamous species, either polygynous or polyandrous, as can be the case in reed warblers [36, 37].

Given that sex-ratios of reed warblers and other common species typically vary inter-annually depending on environmental conditions [37], the use of average, maximum and minimum values between breeding seasons provides an approximate range of plausible population sizes in species with male-biased detectability. However, sex-ratio determination is logistically challenging because it requires intensive sampling by capturing and sexing individuals during the breeding season, which often relies on molecular sexing in monomorphic species [38]. As a consequence, a balanced sex-ratio for monogamous species is generally assumed when no species-specific information is available on particular areas and breeding seasons, despite evidence suggesting that skewed sex-ratios are common in wild bird populations [38]. This assumption may bias population size estimates to an unknown degree and sign depending on the actual sex-ratio of the target population. While estimates considering species-specific sex-ratios according to the literature can be adopted for population size estimates, and indeed subsequently corrected with empirical data on particular populations, regions and breeding seasons, those based on male-biased detectability but not adjusted for sex-ratio (either previously assumed or estimated from field data) clearly strongly underestimate population size. Therefore, obtaining data on population sex-ratio improves population size estimates, especially when based on sampling data with detectability that is typically male-biased during the breeding season.

Any approach for estimating abundance/density from real field data faces a trade-off between sampling effort, which determines the generality of the study and is constrained by budget and logistics, and reliance on assumptions, which, if violated, may result in biased estimates [9]. Therefore, it is important to highlight key assumptions of our approach, and to note that violations of these assumptions, both in this study and in future applications of the method, may have important implications. First, we assume that male vocalization rate is constant within the survey period. As mentioned previously, most male warblers were initially detected based on territorial/sexual vocalizations. Because transects were surveyed from sunrise to midday, variation in singing rate within this period could lead to variation in detection probability among transects (or sections thereof) surveyed in different periods (i.e. sunrise vs. mid-morning vs. midday), and as a result transects surveyed at times of lower vocalization rates will have negatively biased abundance estimates.

Population size estimates based on statistical inference can greatly benefit from the prior validation of the estimation methods by comparison with population size obtained through targeted counts of particular species in selected reference areas [4, 5, 39–41]. In practice, this validation is rarely attained because performing a complete census aimed to record all individuals is often very challenging or logistically demanding, if not altogether unfeasible in very large reference areas hosting large numbers of individuals of the target species [9]. As a consequence, the accuracy of hypothesized estimates of population size of birds based on statistical inference has been often open to question [4, 5, 41]. An approach based on an intensive and focused search of individuals of the target species in relatively small areas and particular habitats can be, however, assumed for many species in reference areas whose intensive monitoring can be logistically affordable [8]. In addition, intensive sampling focused on the direct monitoring of entire populations can allow an *a posteriori* testing of hypothesized estimates derived from inference-rich methods [4–5]. In particular, this intensive approach can be used as a validity test of the population sizes estimated by transforming relative abundance through extrapolation by habitat. For instance, the large population size estimates of reed warblers proposed by using habitat extrapolation [30] were calculated by considering that riverine woodlands, marshlands, rice fields, irrigated crops and suburban areas present the highest warbler densities, thus representing the most favourable habitats for these species; these and other habitat-related densities were extrapolated by habitat to propose national and regional estimates [30]. Strikingly, these habitats mostly correspond to the matrix of unavailable habitat for reed warblers in which suitable and unsuitable reedbed were generally embedded [19, 42]. Therefore, population sizes may be clearly inflated by extrapolating densities to unavailable habitats covering large areas (e.g. irrigated crops and suburban areas in the study area). Because sampling censuses during the breeding season mostly record territorial males of these and other species of small passerines, the overestimated population size from habitat extrapolation may have been even more inflated if it had been corrected by population sex-ratio.

In conclusion, this study highlights the importance of considering the habitat actually used and its suitability, forms of sex-related detection and their interactions in population estimates of common species. Ideally, the value of predictive methods to estimate population size of common species should be tested before conducting large-scale monitoring, rather than *a posteriori*. Although logistically challenging, this can be achieved by designing monitoring programs including intensive censuses of the actual absolute abundance of common species in ad hoc reference areas of variable size, in order to assess and calibrate potential errors and especially to validate predictive models by comparing predicted with actual population size. Our approach combining direct counts of territorial males in suitable habitat and population sex ratio can be a proper alternative to inference-rich predictive modelling based on imperfect habitat-extrapolation of densities of reed warblers and other habitat-specialist species at large spatial scales.

## Supporting information

S1 Table Counts of territorial (singing) male Eurasian reed warblers (*A*. *scirpaceus*) and Great reed warblers (*A*. *arundinaceus*). Censused localities and their UTM coordinates (initial and final coordinates for lotic water stretches, and a central coordinate for localities corresponding to lentic water) are provided according to ED 50 datum. The type of marshland, the mean width (in metres) and the extension (in hectares) of the reedbeds in each locality are also shown.
(DOCX)
