# Supplementary material for: Influence of habitat suitability and sex-related detectability on density and population size estimates of habitat-specialist warblers
Source: PLoS One. 2018 Jul 30;13(7):e0201482. doi: 10.1371/journal.pone.0201482 (PMC6066240; doi:10.1371/journal.pone.0201482)
Supplement: S1 Table — Censused localities and their UTM coordinates (initial and final coordinates for lotic water stretches, and a central coordinate for localities corresponding to lentic water) are provided according to ED 50 datum. The type of marshland, the mean width (in metres) and the extension (in hectares) of the reedbeds in each locality are also shown. (DOCX) [file pone.0201482.s001.docx]

S1 Table

|  |  |  |  |  |  | Counts of territorial (singing) males | |
| --- | --- | --- | --- | --- | --- | --- | --- |
| Site | initial UTM (X/Y) | final UTM (X/Y) | Marshland type | Reedbed width (m) | area (ha) | *A.scirpaceus* | *A.arundinaceus* |
| Arroyo Camarmilla | 468030/4488262 | 467844/4485464 | stream | 3.74 | 1.10 | 7 | 0 |
| Arroyo Carcavillas | 459933/4442847 | 457755/4444479 | stream | 26 | 7.43 | 3 | 0 |
| Arroyo Cavina | 446012/4424961 | 445673/4424286 | stream | 24.56 | 3.59 | 3 | 0 |
| Arroyo Congosto | 452124/4463666 | 451486/4464124 | stream | 12.45 | 1.08 | 7 | 0 |
| Arroyo Culebro | 437782/4458245 | 450267/4462075 | stream | 5.33 | 5.12 | 25 | 2 |
| Arroyo de la Cañada del Pastor Villaconejos | 455825/4436053 | 456272/4437045 | stream | 13.26 | 1.33 | 3 | 1 |
| Arroyo de la Cañada Valdemoro | 445975/4448023 | 450733/4443285 | stream | 4.77 | 3.34 | 7 | 1 |
| Arroyo de la Dehesa | 418055/4462042 | 418717/4461599 | stream | 3.29 | 0.27 | 0 | 0 |
| Arroyo de la Estacada Valle de San Juan | 460676/4434799 | 461740/4437002 | stream | 9.3 | 2.33 | 1 | 0 |
| Arroyo de la Recomba | 432876/4463908 | 432403/4464380 | stream | 0.96 | 0.07 | 0 | 0 |
| Arroyo de la Veguilla | 469389/4446118 | 471728/4452268 | stream | 1.95 | 1.39 | 0 | 0 |
| Arroyo de las Carcavas | 459784/4446833 | 460377/4446912 | stream | 1.74 | 0.12 | 0 | 0 |
| Arroyo de las Salinas | 445746/4429989 | 446423/4429261 | stream | 21.27 | 2.13 | 2 | 0 |
| Arroyo de los Vegones | 418956/4453130 | 419503/4452988 | stream | 2.41 | 0.14 | 0 | 0 |
| Arroyo de Valdezarza | 457946/4444138 | 458217/4443707 | stream | 15.63 | 0.83 | 0 | 0 |
| Arroyo Fuentemaria | 487130/4438708 | 488941/4437582 | stream | 5.99 | 1.46 | 2 | 0 |
| Arroyo Grande | 404782/4461122 | 397833/4464790 | stream | 1.39 | 1.25 | 12 | 0 |
| Arroyo Guaten | 434342/4443499 | 433588/4452526 | stream | 5.45 | 7.55 | 38 | 7 |
| Arroyo Hoyo del Moro | 451866/4431064 | 451767/4430589 | stream | 20.87 | 1.06 | 4 | 0 |
| Arroyo Los Migueles | 454335/4465439 | 453246/4466898 | stream | 8.71 | 1.74 | 2 | 0 |
| Arroyo Martin Roman | 431086/4420043 | 429601/4420894 | stream | 43.28 | 16.14 | 24 | 4 |
| Arroyo Pantueña | 459004/4468244 | 459770/4469150 | stream | 4.78 | 0.66 | 3 | 0 |
| Arroyo Quijorna | 408407/4475035 | 409322/4475347 | stream | 10 | 1.00 | 1 | 0 |
| Arroyo Urb. Pinares Llanos | 423159/4465611 | 423304/4465726 | stream | 3.1 | 0.06 | 0 | 0 |
| Arroyo Vallermoso | 486104/4438009 | 486774/4436563 | stream | 0.43 | 0.08 | 0 | 0 |
| Arroyo Valtarroso | 463840/4434252 | 463734/4435034 | stream | 2.42 | 0.30 | 0 | 0 |
| Balsa-Canal del Jarama Ciempozuelos | 450045/4448379 |  | lake | 7.03 | 0.14 | 1 | 0 |
| Balsas riego San Fernando | 457318/4475163 | 457141/4476388 | lake | 6.03 | 0.37 | 3 | 0 |
| Canal del Jarama Ciempozuelos | 449044/4446794 | 450015/4447247 | stream | 6.69 | 1.52 | 4 | 0 |
| Canal desagüe nave INDITEX | 472126/4486577 |  | lake | 1.2 | 0.03 | 2 | 0 |
| Casa Quintana | 440452/4426339 | 440275/4425875 | lake | 6.18 | 0.47 | 2 | 0 |
| Casa Villamejor | 432991/4421389 | 433837/4421737 | lake | 1.57 | 0.17 | 6 | 2 |
| Caserío del Henares | 459296/4474650 |  | lake | 6.17 | 2.64 | 78 | 11 |
| Caz de Titulcia | 451115/4442472 | 450366/4442155 | stream | 2.82 | 0.42 | 4 | 0 |
| Charca INTA | 463687/4481663 |  | lake | 6.33 | 0.22 | 2 | 1 |
| Charca Navas del Rey | 395042/4471113 |  | lake | 4.53 | 0.29 | 0 | 0 |
| El Porcal. Laguna 1 | 456152/4462137 |  | lake | 8.94 | 2.13 | 7 | 0 |
| El Porcal. Laguna 2 | 455636/4461266 |  | lake | 7.57 | 4.64 | 17 | 6 |
| El Porcal. Laguna 3 | 454784/4460895 |  | lake | 3.98 | 2.51 | 16 | 0 |
| El Porcal. Laguna 4 | 455379/4461626 |  | lake | 3.71 | 1.49 | 23 | 4 |
| El Porcal. Laguna 5 | 455890/4462051 |  | lake | 29.6 | 7.28 | 48 | 15 |
| El Porcal. Laguna 6 | 456474/4461959 |  | lake | 0.97 | 0.21 | 0 | 0 |
| El Porcal. Laguna 7 | 455940/4461722 |  | lake | 2.44 | 0.41 | 0 | 0 |
| El Sotillo | 456916/4468398 | 456827/4468754 | lake | 5.73 | 2.20 | 28 | 12 |
| Embalse de Gozquez | 449138/4454371 |  | lake | 20.6 | 2.39 | 11 | 2 |
| Embalse de los Arroyos | 406981/4480070 |  | lake | 1.39 | 0.24 | 0 | 0 |
| Embalse de los Penascales | 423899/4491890 |  | lake | 0.09 | 0.01 | 0 | 0 |
| Embalse del Aulencia Colmenarejo | 413236/4485709 |  | lake | 2.92 | 0.28 | 1 | 0 |
| Gravera 1 Aldea del Fresno | 396898/4463470 |  | lake | 6.56 | 0.25 | 4 | 0 |
| Gravera 2 Aldea del Fresno | 395893/4460764 |  | lake | 0.1 | 0.01 | 0 | 0 |
| Gravera Atalayuela de Algete El Vado | 452294/4497492 |  | lake | 2.14 | 0.41 | 0 | 2 |
| Gravera Camino de Salomon | 453159/4497915 |  | lake | 7.18 | 0.54 | 0 | 5 |
| Gravera de Talamanca de Jarama | 456274/4507946 |  | lake | 5.11 | 2.11 | 10 | 1 |
| Gravera Molino del Rey | 449110/4446980 |  | lake | 1.71 | 0.09 | 2 | 0 |
| Gravera Talamanca Torrelaguna | 456420/4512615 |  | lake | 9.92 | 0.99 | 3 | 1 |
| Graveras La Aldehuela | 450264/4461768 | 450667/4461890 | lake | 3.85 | 0.17 | 9 | 1 |
| Infantas | 440445/4427760 | 438867/4427064 | lake | 25.7 | 7.30 | 11 | 5 |
| Isla del Herrero | 453697/4455004 |  | lake | 2.61 | 0.47 | 8 | 2 |
| La Asperilla | 456811/4461353 | 457744/4461368 | lake | 2.28 | 1.41 | 32 | 2 |
| La Guindalera | 457471/4473159 | 457616/4472914 | lake | 37.8 | 1.89 | 3 | 0 |
| La Poveda | 460113/4463651 |  | lake | 32.5 | 2.21 | 4 | 0 |
| Lago parque Polvoranca | 431863/4464394 |  | lake | 0 | 0.00 | 0 | 0 |
| Laguna de Casasola | 458798/4445821 |  | lake | 36.96 | 2.07 | 10 | 0 |
| Laguna de Meco | 472195/4486292 |  | lake | 3.07 | 0.31 | 6 | 6 |
| Laguna de Perales | 444041/4462164 | 443864/4462300 | lake | 4.82 | 0.36 | 10 | 2 |
| Laguna de San Galindo | 459583/4447228 |  | lake | 39.12 | 1.96 | 3 | 2 |
| Laguna de San Juan | 455808/4443747 |  | lake | 74.03 | 17.25 | 50 | 4 |
| Laguna del Arroyo Guatel Segundo | 407409/4501026 |  | lake | 0 | 0.00 | 0 | 0 |
| Laguna del Campillo | 457347/4463450 |  | lake | 8.98 | 4.14 | 40 | 9 |
| Laguna Duque de Alburquerque | 454196/4494556 |  | lake | 9.25 | 1.42 | 5 | 6 |
| Laguna El Soto Mostoles | 423385/4464548 |  | lake | 0.1 | 0.01 | 0 | 0 |
| Laguna Junta de los Ríos | 455101/4462678 |  | lake | 5.81 | 0.96 | 11 | 5 |
| Laguna Miralrío | 459205/4467102 | 458949/4467172 | lake | 25.4 | 5.94 | 27 | 3 |
| Laguna Parque S.M.Vega | 451995/4451921 | 452024/4452292 | lake | 0.41 | 0.05 | 4 | 0 |
| Laguna Polígono Velilla | 457813/4468313 | 457676/4468388 | lake | 4.83 | 1.06 | 11 | 0 |
| Laguna Soto de Mozanaque | 452517/4495888 |  | lake | 10.81 | 1.24 | 7 | 4 |
| Lagunas al Sur del ILA | 457794/4473116 | 458087/4473528 | lake | 3.53 | 0.32 | 13 | 1 |
| Lagunas de la Presa del Henares | 457616/4473023 | 458973/4473910 | lake | 7.58 | 1.14 | 1 | 6 |
| Las Arriadas Soto del Parral | 450960/4443846 | 451739/4444725 | lake | 5.12 | 1.77 | 16 | 6 |
| Las Madres | 456626/4461208 | 456686/4461113 | lake | 4.89 | 1.02 | 15 | 1 |
| Las Minas y otros | 453444/4452808 | 452671/4451885 | lake | 26.38 | 6.44 | 39 | 6 |
| Los Ángeles | 454179/4456292 |  | lake | 6.33 | 0.61 | 9 | 1 |
| Mar Chico con Mar Ontigola | 448675/4430055 | 449606/4429422 | lake | 16.64 | 4.08 | 4 | 0 |
| Mar Ontigola | 448675/4430055 | 449606/4429422 | lake | 52.08 | 12.76 | 48 | 6 |
| Picón de los Conejos | 457447/4468316 | 457332/4468427 | lake | 10.75 | 3.36 | 38 | 7 |
| Río Alberche1 | 393569/4458117 | 389771/4452862 | river | 0 | 0.00 | 0 | 0 |
| Río Alberche2 | 393604/4458093 | 397321/4463562 | river | 0 | 0.00 | 0 | 0 |
| Río Algodor | 425833/4417517 | 425324/4417973 | stream | 26.02 | 2.08 | 11 | 0 |
| Río Guadarrama1 | 419018/4449973 | 419498/4452516 | river | 3.23 | 0.97 | 3 | 1 |
| Río Guadarrama2 | 419498/4452516 | 419280/4457111 | river | 0.59 | 0.24 | 3 | 0 |
| Río Guadarrama3 | 419280/4457111 | 419970/4461288 | river | 1.72 | 0.78 | 6 | 0 |
| Río Guadarrama4 | 420093/4461539 | 419598/4469291 | river | 0.19 | 0.15 | 2 | 0 |
| Río Guadarrama5 | 419569/4469080 | 419815/4472158 | river | 2.33 | 0.81 | 20 | 0 |
| Río Guadarrama6 | 419815/4472158 | 419453/4474162 | river | 0 | 0.00 | 0 | 0 |
| Río Guadarrama7 | 419453/4474162 | 419980/4478926 | river | 0.46 | 0.25 | 1 | 0 |
| Río Guadarrama8 | 419980/4478926 | 420602/4481952 | river | 0.46 | 0.19 | 1 | 0 |
| Río Guadarrama9 | 420586/4481955 | 420300/4487049 | river | 0.03 | 0.02 | 0 | 0 |
| Río Henares1 | 457616/4473023 | 458973/4473910 | river | 7.16 | 1.30 | 2 | 0 |
| Río Henares10 | 470534/4481335 | 469455/4480704 | river | 1.78 | 0.39 | 5 | 0 |
| Río Henares11 | 471749/4482815 | 470558/4481352 | river | 1.77 | 0.45 | 0 | 0 |
| Río Henares12 | 471763/4483294 | 471717/4482099 | river | 1.76 | 0.21 | 0 | 0 |
| Río Henares13 | 472418/4483634 | 471768/4483307 | river | 3.76 | 0.25 | 0 | 0 |
| Río Henares14 | 474023/4484850 | 472342/4483581 | river | 2.91 | 0.96 | 4 | 0 |
| Río Henares15 | 474023/4484850 | 474603/4484752 | river | 7.48 | 0.41 | 3 | 0 |
| Río Henares16 | 474582/4484708 | 475452/4485382 | river | 3.73 | 0.45 | 2 | 1 |
| Río Henares17 | 477189/4487418 | 475844/4485329 | river | 5.23 | 1.45 | 2 | 1 |
| Río Henares2 | 459258/4474297 | 460247/4475723 | river | 5.36 | 1.44 | 3 | 2 |
| Río Henares3 | 461143/4476692 | 460176/4475795 | river | 5.04 | 1.17 | 7 | 1 |
| Río Henares4 | 464710/4479628 | 461162/4476696 | river | 3.54 | 1.95 | 3 | 3 |
| Río Henares5 | 465937/4479761 | 464762/4479664 | river | 2.53 | 0.41 | 0 | 0 |
| Río Henares6 | 466776/4480522 | 465848/4479740 | river | 2.17 | 0.27 | 2 | 0 |
| Río Henares7 | 466781/4480528 | 467827/4480082 | river | 0.89 | 0.12 | 0 | 0 |
| Río Henares8 | 469167/4479559 | 467827/4480082 | river | 5.57 | 1.10 | 7 | 0 |
| Río Henares9 | 469175/4479586 | 469435/4480740 | river | 2.68 | 0.56 | 1 | 0 |
| Río Jarama1 | 444543/4431415 | 446080/4433859 | river | 6.67 | 3.00 | 10 | 1 |
| Río Jarama10 | 454123/4461602 | 456215/4463452 | river | 9.88 | 3.31 | 15 | 1 |
| Río Jarama11 | 456215/4463452 | 459948/4465363 | river | 8.86 | 3.90 | 27 | 3 |
| Río Jarama12 | 459948/4465365 | 456631/4469236 | river | 5.14 | 4.63 | 13 | 1 |
| Río Jarama13 | 457465/4472832 | 456631/4469236 | river | 5.98 | 2.99 | 8 | 1 |
| Río Jarama14 | 457465/4472832 | 456586/4473500 | river | 3.14 | 0.63 | 1 | 0 |
| Río Jarama15 | 456586/4473500 | 454959/4477078 | river | 3.75 | 2.62 | 5 | 0 |
| Río Jarama16 | 453506/4483053 | 454857/4477518 | river | 4.4 | 3.06 | 9 | 0 |
| Río Jarama17 | 452824/4488593 | 453511/4483120 | river | 7.23 | 5.11 | 23 | 4 |
| Río Jarama18 | 452836/4488295 | 451799/4492604 | river | 5.03 | 2.71 | 19 | 1 |
| Río Jarama19 | 451799/4492604 | 453035/4499042 | river | 5.61 | 5.24 | 36 | 6 |
| Río Jarama2 | 446080/4433859 | 448170/4437508 | river | 10.53 | 7.90 | 12 | 1 |
| Río Jarama20 | 453148/4503709 | 453016/4498979 | river | 2.35 | 1.51 | 5 | 4 |
| Río Jarama21 | 453148/4503709 | 455924/4509098 | river | 3.94 | 3.51 | 36 | 10 |
| Río Jarama22 | 456387/4513596 | 455924/4509098 | river | 1.86 | 1.20 | 13 | 1 |
| Río Jarama23 | 456357/4513672 | 458966/4518077 | river | 0.86 | 0.60 | 4 | 0 |
| Río Jarama24 | 458966/4518077 | 460128/4521222 | river | 0.87 | 0.35 | 0 | 0 |
| Río Jarama25 | 460128/4521222 | 460196/4523528 | river | 0.96 | 0.28 | 0 | 0 |
| Río Jarama26 | 460196/4523528 | 462506/4525114 | river | 0.64 | 0.19 | 0 | 0 |
| Río Jarama3 | 448170/4437508 | 450386/4442147 | river | 15.03 | 13.53 | 23 | 1 |
| Río Jarama4 | 450615/4443178 | 452189/4445920 | river | 2.76 | 1.38 | 25 | 2 |
| Río Jarama5 | 452189/4445920 | 452471/4449308 | river | 6.18 | 2.47 | 18 | 1 |
| Río Jarama6 | 452512/4449206 | 452163/4450957 | river | 2.5 | 0.62 | 7 | 1 |
| Río Jarama7 | 453570/4454285 | 452163/4450957 | river | 7.65 | 4.21 | 30 | 4 |
| Río Jarama8 | 453570/4458285 | 454105/4453904 | river | 5.32 | 2.92 | 21 | 1 |
| Río Jarama9 | 453570/4458285 | 454123/4461602 | river | 5.05 | 3.03 | 15 | 2 |
| Río Manzanares1 | 452850/4463859 | 454123/4461602 | river | 3.9 | 3.51 | 6 | 1 |
| Río Manzanares2 | 447344/4462486 | 452850/4463859 | river | 2.86 | 2.28 | 8 | 0 |
| Río Manzanares3 | 445487/4464039 | 447344/4462486 | river | 0.97 | 0.72 | 3 | 0 |
| Río Manzanares4 | 443778/4465493 | 445487/4464039 | river | 0.85 | 0.55 | 1 | 0 |
| Río Manzanares5 | 441523/4470165 | 443748/4465540 | river | 0.83 | 0.43 | 1 | 0 |
| Río Manzanares6 | 436000/4482007 | 436713/4478963 | river | 0.34 | 0.12 | 1 | 0 |
| Río Manzanares7 | 433959/4485852 | 436000/4482007 | river | 3.43 | 1.61 | 4 | 0 |
| Río Manzanares8 | 433959/4485852 | 433276/4487581 | river | 3.48 | 0.90 | 8 | 0 |
| Río Perales | 403511/4469428 | 397791/4464748 | stream | 0.08 | 0.09 | 0 | 0 |
| Río Salado | 491939/4443627 | 493908/4441826 | stream | 0 | 0.00 | 0 | 0 |
| Río Tajo1 | 492261/4445655 | 494153/4445164 | river | 1.69 | 0.43 | 9 | 3 |
| Río Tajo10 | 480521/4435083 | 481790/4435843 | river | 5.04 | 0.86 | 11 | 1 |
| Río Tajo11 | 480523/4435049 | 476966/4433749 | river | 5.72 | 3.42 | 76 | 14 |
| Río Tajo12 | 476963/4433774 | 474836/4433548 | river | 3.11 | 1.12 | 16 | 7 |
| Río Tajo13 | 472629/4432582 | 474836/4433548 | river | 8.4 | 4.66 | 32 | 9 |
| Río Tajo14 | 470624/4432924 | 467916/4433353 | river | 3.19 | 1.75 | 19 | 8 |
| Río Tajo15 | 463577/4432784 | 467897/4433346 | river | 6.95 | 5.03 | 55 | 4 |
| Río Tajo16 | 463897/4433346 | 460387/4432869 | river | 3.35 | 1.90 | 17 | 3 |
| Río Tajo17 | 456456/4432983 | 460398/4432743 | river | 5.56 | 3.45 | 11 | 2 |
| Río Tajo18 | 456456/4432983 | 454928/4433649 | river | 1.66 | 0.79 | 7 | 0 |
| Río Tajo19 | 453450/4433040 | 454915/4433636 | river | 7.09 | 1.42 | 25 | 3 |
| Río Tajo2 | 492261/4445655 | 492009/4445259 | river | 4.77 | 0.22 | 7 | 0 |
| Río Tajo20 | 451397/4432577 | 453450/4433040 | river | 3.26 | 1.08 | 35 | 1 |
| Río Tajo21 | 451345/4432576 | 448384/4432018 | river | 2.31 | 1.15 | 3 | 1 |
| Río Tajo22 | 444543/4431415 | 448384/4432018 | river | 4.05 | 2.43 | 7 | 2 |
| Río Tajo23 | 443235/4431984 | 444543/4431415 | river | 2.01 | 0.40 | 10 | 1 |
| Río Tajo24 | 439480/4429232 | 443235/4431984 | river | 3.64 | 3.10 | 35 | 5 |
| Río Tajo25 | 439480/4429232 | 436834/4423727 | river | 10.1 | 8.08 | 24 | 3 |
| Río Tajo26 | 436834/4423727 | 431390/4423001 | river | 6.42 | 5.77 | 34 | 2 |
| Río Tajo27 | 431390/4423001 | 427220/4421409 | river | 5.27 | 3.95 | 50 | 0 |
| Río Tajo28 | 427220/4421409 | 425191/4418235 | river | 7.43 | 4.09 | 16 | 1 |
| Río Tajo3 | 492009/4445259 | 491679/4444682 | river | 5.29 | 0.36 | 13 | 1 |
| Río Tajo4 | 491679/4444682 | 491775/4443786 | river | 3.29 | 0.41 | 4 | 1 |
| Río Tajo5 | 491775/4443786 | 489585/4443126 | river | 2.78 | 0.80 | 9 | 1 |
| Río Tajo6 | 489585/4443126 | 487216/4440959 | river | 2.36 | 1.09 | 5 | 0 |
| Río Tajo7 | 487178/4440938 | 486865/4438963 | river | 2.97 | 0.99 | 27 | 0 |
| Río Tajo8 | 482695/4436931 | 486865/4438963 | river | 6.24 | 4.48 | 76 | 24 |
| Río Tajo9 | 482828/4436945 | 481892/4436083 | river | 4.8 | 0.67 | 8 | 1 |
| Río Tajuna1 | 451108/4442446 | 450415/4442176 | river | 4.72 | 0.62 | 1 | 1 |
| Río Tajuna10 | 482860/4459503 | 484623/4463753 | river | 3.68 | 2.46 | 3 | 0 |
| Río Tajuna11 | 484623/4463753 | 487761/4467139 | river | 2.87 | 1.78 | 6 | 0 |
| Río Tajuna2 | 451136/4442448 | 456763/4445101 | river | 5.51 | 6.17 | 22 | 0 |
| Río Tajuna3 | 459610/4447018 | 456763/4445101 | river | 4.82 | 2.49 | 4 | 0 |
| Río Tajuna4 | 459610/4447018 | 460282/4449491 | river | 4.79 | 2.02 | 10 | 0 |
| Río Tajuna5 | 460286/4449485 | 464813/4452765 | river | 3.5 | 3.21 | 5 | 0 |
| Río Tajuna6 | 464813/4452765 | 471279/4453148 | river | 3.81 | 3.23 | 2 | 0 |
| Río Tajuna7 | 471279/4453168 | 476244/4455012 | river | 4.82 | 3.85 | 17 | 0 |
| Río Tajuna8 | 479961/4455455 | 476244/4455012 | river | 4.14 | 2.41 | 12 | 0 |
| Río Tajuna9 | 480050/4455389 | 482870/4459499 | river | 4.34 | 3.01 | 1 | 0 |
| Río Torote1 | 464762/4479664 | 463963/4481227 | river | 2.64 | 0.48 | 3 | 0 |
| Río Torote2 | 463910/4482719 | 463963/4481227 | river | 1.83 | 0.41 | 2 | 0 |
| Río Torote3 | 463910/4482719 | 464145/4485370 | river | 1.85 | 0.57 | 0 | 0 |
| Río Torote4 | 464494/4488234 | 464035/4485175 | river | 2.77 | 1.12 | 12 | 2 |
| Río Torote5 | 464521/4488248 | 464621/4490542 | river | 3.55 | 1.15 | 17 | 0 |
| Río Torote6 | 464621/4490542 | 466482/4496032 | river | 2.79 | 1.81 | 12 | 0 |
| Río Torote7 | 466512/4496044 | 468923/4499984 | river | 3.3 | 1.98 | 6 | 2 |
| Santa Juliana | 454395/4456306 | 454491/4457150 | lake | 5.04 | 0.76 | 15 | 2 |
| Soto Cisneros La Cigüeña | 456166/4460494 |  | lake | 2.57 | 0.36 | 10 | 0 |
| Soto de las Cuevas | 447791/4438476 |  | lake | 3.81 | 0.54 | 12 | 2 |
| Soto del Lugar | 435138/4422346 | 434208/4423042 | lake | 68.81 | 12.39 | 29 | 3 |
| Soto Gutiérrez N | 452008/4448883 |  | lake | 16.68 | 4.10 | 10 | 2 |
| Soto Gutiérrez S | 451736/4447395 |  | lake | 4.81 | 0.44 | 5 | 2 |
| Soto Juan Antonio | 452984/4451386 |  | lake | 3.44 | 0.51 | 6 | 1 |
| Soto Pajares | 454373/4457470 | 455066/4458839 | lake | 3.42 | 2.58 | 66 | 8 |
| Sotomayor | 454525/4431932 | 454948/4430784 | stream | 14.78 | 1.82 | 7 | 2 |
| Sotomayor Casa Monta | 452630/4432106 | 453263/4431394 | stream | 10.21 | 0.87 | 1 | 0 |
